# Association of SGLT2 inhibitors with cardiovascular, kidney, and safety outcomes among patients with diabetic kidney disease: a meta-analysis

**DOI:** 10.1186/s12933-022-01476-x

**Published:** 2022-03-23

**Authors:** Arnaud D. Kaze, Min Zhuo, Seoyoung C. Kim, Elisabetta Patorno, Julie M. Paik

**Affiliations:** 1 Department of Medicine, LifePoint Health, Danville, VA USA; 2 Division of Pharmacoepidemiology and Pharmacoeconomics, Department of Medicine, Brigham and Women’s Hospital, 1620 Tremont Street, Suite 3030, Boston, MA USA; 3 Harvard Medical School, Boston, MA USA; 4 Division of Renal (Kidney) Medicine, Department of Medicine, Brigham and Women’s Hospital, Boston, MA USA; 5 Division of Nephrology, Department of Medicine, Beth Israel Deaconess Medical Center, Boston, MA USA; 6 New England Geriatric Research Education and Clinical Center, VA Boston Healthcare System, Boston, MA USA

**Keywords:** SGLT2 inhibitors, Diabetic kidney disease, Cardiovascular outcomes, Kidney outcomes, Safety, Meta-analysis

## Abstract

**Background:**

We conducted a systematic review and meta-analysis of the cardiovascular, kidney, and safety outcomes of sodium-glucose cotransporter 2 inhibitors (SGLT2i) among patients with diabetic kidney disease (DKD).

**Methods:**

We searched electronic databases for major randomized placebo-controlled clinical trials published up to September 30, 2021 and reporting on cardiovascular and kidney outcomes of SGLT2i in patients with DKD. DKD was defined as chronic kidney disease in individuals with type 2 diabetes. Random-effects meta-analysis models were used to estimate pooled hazard ratios (HR) and 95% confidence intervals (CI) for clinical outcomes including major adverse cardiovascular events (MACE: myocardial infarction [MI], stroke, and cardiovascular death), kidney composite outcomes (a combination of worsening kidney function, end-stage kidney disease, or death from renal or cardiovascular causes), hospitalizations for heart failure (HHF), deaths and safety events (mycotic infections, diabetic ketoacidosis [DKA], volume depletion, amputations, fractures, urinary tract infections [UTI], acute kidney injury [AKI], and hyperkalemia).

**Results:**

A total of 26,106 participants with DKD from 8 large-scale trials were included (median age: 65.2 years, 29.7–41.8% women, 53.2–93.2% White, median follow-up: 2.5 years). SGLT2i were associated with reduced risks of MACE (HR 0.83, 95% CI 0.75–0.93), kidney composite outcomes (HR 0.66, 95% CI 0.58–0.75), HHF (HR 0.62, 95% CI 0.55–0.71), cardiovascular death (HR 0.84, 95% CI 0.74–0.96), MI (HR 0.78, 95% CI 0.67–0.92), stroke (HR 0.76, 95% CI 0.59–0.97), and all-cause death (HR 0.86, 95% CI 0.77–0.96), with no significant heterogeneity detected. Similar results were observed among participants with reduced estimated glomerular filtration rate (eGFR: < 60 mL/min/1.73m^2^). The relative risks (95% CI) for adverse events were 3.89 (1.42–10.62) and 2.50 (1.32–4.72) for mycotic infections in men and women respectively, 3.54 (0.82–15.39) for DKA, and 1.29 (1.13–1.48) for volume depletion.

**Conclusions:**

Among adults with DKD, SGLT2i were associated with reduced risks of MACE, kidney outcomes, HHF, and death. With a few exceptions of more clear safety signals, we found overall limited data on the associations between SGLT2i and safety outcomes. More research is needed on the safety profile of SGLT2i in this population.

**Supplementary Information:**

The online version contains supplementary material available at 10.1186/s12933-022-01476-x.

## Background

Type 2 diabetes (T2D) remains highly common in the United States [1]. Nearly 32 million Americans currently live with T2D and its prevalence is expected to rise [2, 3]. Diabetes-related microvascular complications such as diabetic kidney disease (DKD) constitute a significant public health problem. Indeed, nearly 40% of patients with T2D develop DKD [4]. DKD is a leading cause of chronic kidney disease (CKD) and end-stage kidney disease (ESKD) [4]. Furthermore, most of the excess mortality risk observed in patients with diabetes may be related to the presence of DKD [5].

Sodium-glucose cotransporter 2 inhibitors (SGLT2i) have recently emerged as a new class of oral glucose-lowering agents with pleiotropic effects including reduction in cardiovascular and kidney outcomes among patients with T2D [6–11]. SGLT2i prescriptions have steadily increased among patients with DKD [12]. However, some uncertainty remains on the effects of SGLT2i along the entire spectrum of DKD. For instance, a relatively small number of participants reached ESKD in early clinical trials of SGLT2i [7, 13, 14]; studies of the effects of SGLT2i on clinical outcomes such as stroke among individuals with DKD have yielded inconsistent results [15]. Moreover, the cardiovascular outcome trials focusing on SGLT2i have usually been underpowered to evaluate most adverse events in people with DKD [6–10, 13, 14, 16]. Therefore, we performed a systematic review and meta-analysis of the major cardiovascular and kidney outcomes trials to summarize and update the currently available evidence on the effects of SGLT2i on cardiovascular, kidney and safety outcomes among patients with DKD.

## Methods

This systematic review and meta-analysis was registered with PROSPERO (CRD42021282869)[17] and reported according to the Preferred Reporting Items for Systematic Reviews and Meta-Analyses (PRISMA) guideline [18].

### Search strategy

We conducted a comprehensive search of PubMed and Embase for major randomized, placebo-controlled clinical trials of SGLT2i published up to September 30, 2021 and reporting on cardiovascular and kidney outcomes as well as adverse events among adults with DKD. The SGLT2i included empagliflozin, dapagliflozin, canagliflozin, ertugliflozin, and sotagliflozin. Sotagliflozin is an SGLT2i that also inhibits gastrointestinal sodium glucose cotransporter 1 receptors in the gastrointestinal tract. DKD was defined as the presence of CKD in individuals with T2D, defined as estimated glomerular filtration rate (eGFR) < 60 mL/min/1.73 m^2^ and/or urine albumin-to-creatinine ratio (UACR) ≥ 30 mg/g. Two reviewers (ADK and MZ) independently identified articles and sequentially screened them for inclusion, starting with titles and abstracts, then full-text review. Additionally, reference lists of identified studies were manually scanned, and citing references screened through the ISI Web of Knowledge database, for possible additional eligible studies.

### Eligibility criteria, data extraction and assessment of study quality

Studies were considered eligible if they met all of the following criteria: (1) the study design was a randomized controlled trial (RCT) of cardiovascular or kidney outcomes of SGLT2i; (2) the study reported on cardiovascular, kidney, or adverse events of SGLT2i among individuals with DKD; (3) the hazard ratio (HR) or relative risk (RR) and its corresponding 95% confidence interval (CI) or enough data to calculate them were reported. We excluded studies in people without diabetes or CKD. Two investigators (ADK and MZ) independently abstracted data from eligible studies and conducted quality assessment. Data were extracted from the primary trials’ reports as well as from secondary data analyses where available [15, 19–25]. We assessed the quality of studies using the Cochrane Risk of Bias tool [26].

### Outcomes

The efficacy outcomes assessed in this meta-analysis include: (1) major adverse cardiovascular events (MACE) defined as a composite of nonfatal myocardial infarction (MI), nonfatal stroke, or cardiovascular death; (2) hospitalization for HF (HHF); (3) cardiovascular death; (4) fatal and nonfatal MI; (5) fatal and nonfatal stroke; (6) all‐cause mortality; and (7) kidney composite outcomes. The kidney composite outcome definitions varied across trials (Additional file 1: Table S1) but generally included a combination of worsening eGFR or serum creatinine, ESKD with or without renal replacement therapy or transplant, or death from renal or cardiovascular causes.

We evaluated the safety outcomes that have been reported across the clinical trials including mycotic infections, diabetic ketoacidosis (DKA), volume depletion, amputations, fractures, urinary tract infections (UTI), acute kidney injury (AKI), and hyperkalemia.

### Statistical analysis

For this meta-analysis, we used published data; and not individual participant-level data. For each outcome, we sought to identify HRs and 95%CIs of the effects of SGLT2i in participants with DKD from individual studies. As HRs were not always available for adverse events, we used in order of preference HRs, then rate ratios or risk ratios to maximize the use of trial‐level data. The pooled effect estimates and associated 95% CI were computed using random-effects meta-analysis models. The random-effects model is the most conservative approach as it makes allowances for within and between-study heterogeneity [27]. For each outcome, the HR and its 95% CI limits were logarithmically transformed before the meta-analysis. The meta-analysis was implemented on the natural logarithmic scale, with results exponentiated and reported on the original HR scale. The z statistic was used to test the null hypothesis (that the SGLT2i is not associated with the outcome). We assessed the heterogeneity between studies using Cochran’s Q statistic, and *I*^2^ statistics [28, 29]. Publication bias was assessed by the Egger’s test [30]. Furthermore, we performed subgroup analyses stratified by eGFR range and albuminuria levels. Albuminuria levels were classified as moderate albuminuria (UACR 30 – 300 mg/g) or severe albuminuria (UACR ≥ 300 mg/g).

A two-sided *P*-value was deemed statistically significant. All analyses were performed using Stata software (Stata Corp V.14, Texas, USA).

## Results

### Characteristics of studies

The study selection process is summarized in Additional file 1: Fig. S1. A total of 8 placebo-controlled trials evaluating 5 SGLT2i were included (Additional file 1: Table S2). These included the EMPA‐REG OUTCOME (Empagliflozin Cardiovascular Outcome Event Trial in Type 2 diabetes Mellitus Patients) [6], CANVAS (Canagliflozin Cardiovascular Assessment Study) Program [11], DECLARE‐TIMI 58 (Dapagliflozin Effect on Cardiovascular Events–Thrombolysis in Myocardial Infarction 58) [7], CREDENCE (Canagliflozin and Renal Events in Diabetes with Established Nephropathy Clinical Evaluation) [8], DAPA-CKD (Dapagliflozin and Prevention of Adverse Outcomes in Chronic Kidney Disease) [9], VERTIS CV (Evaluation of Ertugliflozin Efficacy and Safety Cardiovascular Outcomes Trial) [10], SCORED (Effect of Sotagliflozin on Cardiovascular and Renal Events in Patients with Type 2 Diabetes and Moderate Renal Impairment Who Are at Cardiovascular Risk) [16], and SOLOIST-WHF (Effect of Sotagliflozin on Cardiovascular Events in Patients with Type 2 Diabetes Post Worsening Heart Failure) [31]. All the studies had a low risk of bias (Additional file 1: Table S3) [26].

We included data from 26,106 participants, with a median follow-up of 2.5 years (interquartile range: 1.9–3.1). Across the 8 trials, the mean age of participants ranged from 61.8 to 69 years, the proportion of women from 29.7 to 41.8%; White individuals made up the majority of participants (53.2–93.2%). The mean HbA_1C_ ranged from 7.1 to 8.3%. The proportion of patients with a history of established CVD at baseline varied from 37.4 to 100%.

### Association of SGLT2i with cardiovascular and kidney outcomes among patients with diabetic kidney disease

**Fig. 1:**
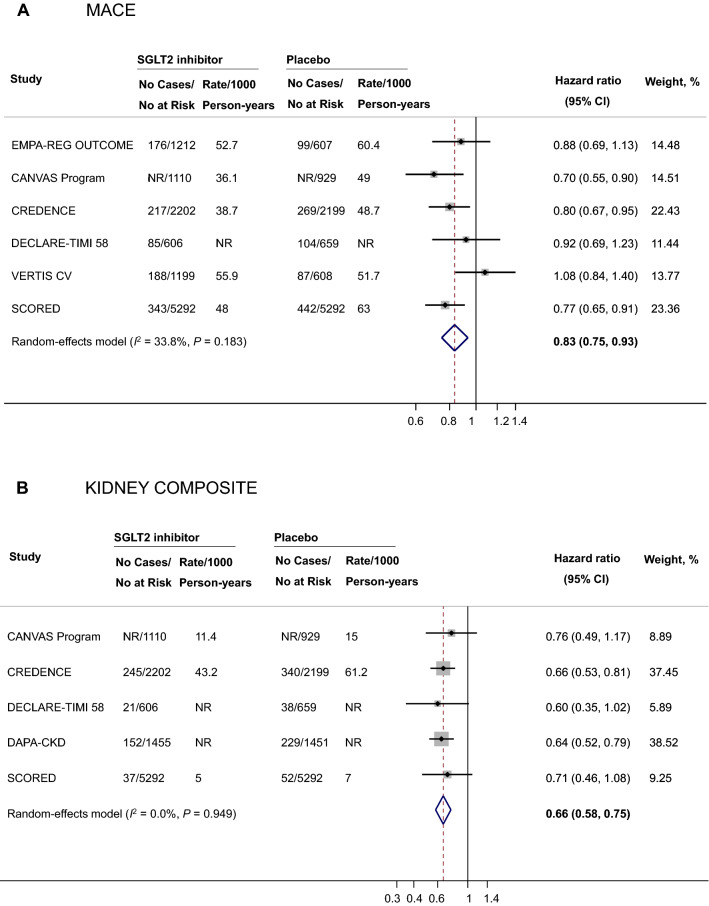
Effects of SGLT2 inhibitors on major adverse cardiovascular events (**A**) and kidney composite outcomes (**B**) among individuals with diabetic kidney disease. CANVAS indicates Canagliflozin Cardiovascular Assessment Study, *CI* confidence interval, *CREDENCE* Canagliflozin and Renal Events in Diabetes with Established Nephropathy Clinical Evaluation, *DECLARE‐TIMI 58* Dapagliflozin Effect on Cardiovascular Events–Thrombolysis in Myocardial Infarction 58, *EMPA‐REG OUTCOME* Empagliflozin Cardiovascular Outcome Event Trial in Type 2 diabetes Mellitus Patients, *I*^2^ I-squared, *MACE* major adverse cardiovascular event, *NR* not reported, *SCORED* Effect of Sotagliflozin on Cardiovascular and Renal Events in Patients with Type 2 Diabetes and Moderate Renal Impairment Who Are at Cardiovascular Risk, *SGLT2* sodium-glucose cotransporter 2, *VERTIS CV* Evaluation of Ertugliflozin Efficacy and Safety Cardiovascular Outcomes Trial

Meta-analyses for the effect of SGLT2i on clinical outcomes are summarized in Table 1. Overall, 2,271 participants experienced a MACE. The use of SGLT2i was associated with a 17% reduction in the risk of MACE (HR 0.83, 95% CI 0.75–0.93, *I*^2^ = 33.8%, *P* for heterogeneity = 0.183, n = 21,913, Fig. 1). Additionally, SGLT2i were associated with reductions in the risks of HHF (HR 0.62, 95% CI 0.55–0.71, *I*^2^ = 0.0%, *P* for heterogeneity = 0.844, n = 22,346), cardiovascular death (HR 0.84, 95% CI 0.74–0.96, *I*^2^ = 0.0%, *P* for heterogeneity = 0.639, n = 20,539), fatal and nonfatal MI (HR 0.78, 95% CI 0.67–0.92, *I*^2^ = 7.7%, *P* for heterogeneity = 0.363, n = 20,108), fatal and nonfatal stroke (HR 0.76, 95% CI 0.59–0.97, *I*^2^ = 41.3%, *P* for heterogeneity = 0.146, n = 20,108, Additional file 1: Fig. S2), as well as all-cause mortality (HR 0.86, 95% CI 0.77–0.96, *I*^2^ = 14.5%, *P* for heterogeneity = 0.322, n = 21,406).

**Table 1:** Effect of SGLT2 inhibitors on clinical outcomes in adults with diabetic kidney disease

Outcome	No. studies	No. events	Sample size	HR (95% CI)	*I*^2^, %	*P*_Heterogeneity_	*P*_Egger test_
MACE	6	2271	21,913	0.83 (0.75–0.93)	33.8	0.183	0.287
Kidney composite	5	1197	21,195	0.66 (0.58–0.75)	0.0	0.949	0.513
HHF	6	1219	22,346	0.62 (0.55–0.71)	0.0	0.844	0.267
Cardiovascular death	5	953	20,539	0.84 (0.74–0.96)	0.0	0.639	0.996
Fatal and nonfatal MI	5	498*	20,108	0.78 (0.67–0.92)	7.7	0.363	0.671
Fatal and nonfatal stroke	5	332*	20,108	0.76 (0.59–0.97)	41.3	0.146	0.564
All-cause mortality	5	1451	21,406	0.86 (0.77–0.96)	14.5	0.322	0.268

*The number of MI events and stroke cases from the SCORED trial were not reported in the primary trials and are not included in the table. CI indicates confidence interval; *HHF* hospitalization for heart failure, *HR* hazard ratio, *I*^2^, I-squared, *MACE* Major Adverse Cardiovascular Events, *MI* myocardial infarction, *SCORED* Effect of Sotagliflozin on Cardiovascular and Renal Events in Patients with Type 2 Diabetes and Moderate Renal Impairment Who Are at Cardiovascular Risk, *SGLT2* sodium-glucose cotransporter 2; SGLT2, sodium-glucose cotransporter 2, *SOLOIST-WHF* Effect of Sotagliflozin on Cardiovascular Events in Patients with Type 2 Diabetes Post Worsening Heart Failure, *VERTIS CV* Evaluation of Ertugliflozin Efficacy and Safety Cardiovascular Outcomes Trial

A total of 1,197 participants experienced a kidney composite outcome. SGLT2i were associated with a 34% reduction in the risk of a kidney composite outcome (HR 0.66, 95% CI 0.58–0.75, *I*^2^ = 0.0%, *P* for heterogeneity = 0.949, n = 21,195, Fig. 1).

### Association of SGLT2i with cardiovascular and kidney outcomes among participants with reduced eGFR

The meta-analyses of the effects of SGLT2i on clinical outcomes by baseline eGFR are displayed in Table 2. When analyses were restricted to participants with reduced eGFR (eGFR < 60 mL/min/1.73 m^2^) regardless of albuminuria status (n = 20,106), SGLT2i remained associated with lower risks of MACE (HR 0.82, 95% CI 0.74–0.91, *I*^2^ = 8.6%, *P* for heterogeneity = 0.363, n = 20,106), HHF (HR 0.61, 95% CI 0.54–0.70, *I*^2^ = 0.0%, *P* for heterogeneity = 0.740, n = 20,106), cardiovascular death (HR 0.86, 95% CI 0.75–0.98, *I*^2^ = 0.0%, *P* for heterogeneity = 0.912, n = 18,299), nonfatal and fatal MI (HR 0.75, 95% CI 0.63–0.90, *I*^2^ = 0.0%, *P* for heterogeneity = 0.480, n = 15,707), nonfatal and fatal stroke (HR 0.75, 95% CI 0.55–1.01, *I*^2^ = 38.3%, *P* for heterogeneity = 0.151, n = 15,707), and kidney composite outcomes (HR 0.65, 95% CI 0.55–0.78, *I*^2^ = 0.0%, *P* for heterogeneity = 0.645, n = 16,480). There was a trend towards a reduction in all-cause mortality, though confidence intervals included the null hypothesis value (HR 0.93, 95% CI 0.81–1.07, *I*^2^ = 0.0%, *P* for heterogeneity = 0.302, n = 13,668 participants across 3 studies).

**Table 2:** Effect of SGLT2 inhibitors on clinical outcomes among participants with reduced eGFR

Outcome	No. studies	No. events	Sample size	HR (95% CI)	*I*^2^, %	*P*_Heterogeneity_	*P*_Egger test_
Overall (eGFR < 60 mL/min/1.73m^2^)							
MACE	6	2102	20,106	0.82 (0.74–0.91)	8.6	0.363	0.810
Kidney composite	4	530	16,480	0.65 (0.55–0.78)	0.0	0.645	0.771
HHF	6	1125	20,106	0.61 (0.54–0.70)	0.0	0.740	0.099
Cardiovascular death	5	834	18,299	0.86 (0.75–0.98)	0.0	0.912	0.363
Fatal and nonfatal MI	4	320*	15,707	0.75 (0.63–0.90)	0.0	0.480	0.879
Fatal and nonfatal stroke	4	190*	15,707	0.75 (0.55–1.01)	38.3	0.151	0.855
All-cause mortality	3	837	13,668	0.93 (0.81–1.07)	0.0	0.638	0.147
eGFR < 45 mL/min/1.73m^2^**							
MACE	3	347	2437	0.75 (0.60–0.93)	0.0	0.797	0.921
Kidney composite	2	225	1867	0.70 (0.54–0.92)	0.0	0.841	NA
HHF	3	166	2437	0.60 (0.44–0.82)	0.0	0.522	0.193
Cardiovascular death	3	191	2437	0.83 (0.62–1.11)	0.0	0.699	0.925
Fatal and nonfatal MI	2	71	1124	0.70 (0.39–1.26)	28.3	0.238	NA
Fatal and nonfatal stroke	2	38	1124	0.52 (0.23–1.17)	30.2	0.231	NA
All-cause mortality	1	74	570	0.86 (0.54–1.38)	NA	NA	NA

*The number of MI and stroke events were not reported in SCORED and are therefore not included in the table. Likewise, the number of HHF/cardiovascular death events were not reported in SOLOIST-WHF and are not included in the table. CI indicates confidence interval, *eGFR* estimated glomerular filtration rate, *HHF* hospitalization for heart failure, *MACE* major adverse cardiovascular events, *HR* hazard ratio, *MI* myocardial infarction, *NA* not applicable, *NR* not reported, *SCORED* Effect of Sotagliflozin on Cardiovascular and Renal Events in Patients with Type 2 Diabetes and Moderate Renal Impairment Who Are at Cardiovascular Risk, *SGLT2* sodium-glucose cotransporter 2, *SOLOIST-WHF* Effect of Sotagliflozin on Cardiovascular Events in Patients with Type 2 Diabetes Post Worsening Heart Failure

**The lower ends of eGFR ranged from 25 to 30 mL/min/1.73 m^2^

### Association of SGLT2i with cardiovascular and kidney outcomes among participants with moderate or severe albuminuria

The meta-analyses of the effects of SGLT2i on clinical outcomes by baseline albuminuria levels are summarized in Table 3. Among participants with severely increased albuminuria, SGLT2i were associated with reduced risks of MACE (HR 0.73, 95% CI 0.65–0.83, *I*^2^ = 0.0%, *P* for heterogeneity = 0.444, n = 9,216), HHF (HR 0.62, 95% CI 0.51–0.75, *I*^2^ = 0.0%, *P* for heterogeneity = 0.915, n = 6,685), cardiovascular death (HR 0.72, 95% CI 0.59–0.87, *I*^2^ = 0.0%, *P* for heterogeneity = 0.408, n = 5,930), all-cause mortality (HR 0.77, 95% CI 0.65–0.90, *I*^2^ = 0.0%, *P* for heterogeneity = 0.415, n = 5,930), and kidney composite outcomes (HR 0.63, 95% CI 0.53–0.76, *I*^2^ = 0.8%, *P* for heterogeneity = 0.365, n = 8,447). There was a trend towards a reduction in MI (HR 0.81, 95% CI 0.64–1.03, *I*^2^ = 0.0%, *P* for heterogeneity = 0.654, n = 5,930) and stroke (HR 0.85, 95% CI 0.65–1.11, *I*^2^ = 0.0%, *P* for heterogeneity = 0.410, n = 5,930), although confidence intervals included the null value. Among participants with moderately increased albuminuria, the HRs were 0.92 (95% CI 0.79–1.07) for MACE, 0.60 (95% CI 0.47–0.77) for HHF, 0.70 (95% CI 0.35–1.38) for cardiovascular death, 1.01 (95% CI 0.77–1.32) for MI, 1.06 (95% CI 0.76–1.48) for stroke, 0.78 (95% CI 0.47–1.28) for all-cause mortality, and 0.98 (95% CI 0.62–1.57) for kidney composite outcomes (Table 3).

**Table 3:** Effect of SGLT2 inhibitors on clinical outcomes among participants with moderate or severe albuminuria

Outcome	No. studies	No. events	Sample size	HR (95% CI)	*I*^2^, %	*P*_Heterogeneity_	*P*_Egger test_
Overall							
MACE	4	1284*	17,084	0.80 (0.71–0.90)	25.6	0.234	0.683
Kidney composite	4	1124*	17,208	0.66 (0.58–0.75)	1.4	0.407	0.530
HHF	4	670	13,456	0.61 (0.52–0.71)	0.0	0.916	0.791
Cardiovascular death	3	642	10,209	0.70 (0.55–0.88)	50.9	0.086	0.378
Fatal and nonfatal MI	3	503	10,209	0.89 (0.75–1.07)	0.0	0.679	0.355
Fatal and nonfatal stroke	3	382	10,209	0.92 (0.75–1.14)	0.0	0.457	0.279
All-cause mortality	4	817*	13,115	0.76 (0.66–0.88)	28.8	0.219	0.285
Moderate albuminuria							
MACE	3	520*	7868	0.92 (0.79–1.07)	0.0	0.795	0.342
Kidney composite	2	70*	5855	0.98 (0.62–1.57)	0.0	0.962	NA
HHF	3	261	6771	0.60 (0.47–0.77)	0.0	0.473	0.283
Cardiovascular death	2	229	4279	0.70 (0.35–1.38)	84.2	0.012	NA
Fatal and nonfatal MI	2	233	4279	1.01 (0.77–1.32)	0.0	0.774	NA
Fatal and nonfatal stroke	2	153	4279	1.06 (0.76–1.48)	0.0	0.363	NA
All-cause mortality	2	147*	4279	0.78 (0.47–1.28)	80.4	0.024	NA
Severe Albuminuria							
MACE	4	764*	9216	0.73 (0.65–0.83)	0.0	0.444	0.428
Kidney composite	3	673*	8447	0.63 (0.53–0.76)	0.8	0.365	0.729
HHF	4	409	6685	0.62 (0.51–0.75)	0.0	0.915	0.815
Cardiovascular death	3	413	5930	0.72 (0.59–0.87)	0.0	0.408	0.348
Fatal and nonfatal MI	3	270	5930	0.81 (0.64–1.03)	0.0	0.654	0.389
Fatal and nonfatal stroke	3	229	5930	0.85 (0.65–1.11)	0.0	0.410	NA
All-cause mortality	3	473*	5930	0.77 (0.65–0.90)	0.0	0.415	0.234

*The number of MACE and kidney composite outcomes were not reported in SCORED and are therefore not included in the table. Likewise, the number of all-cause deaths by albuminuria were not reported in the CANVAS program and are not included in the table. CANVAS indicates Canagliflozin Cardiovascular Assessment Study, *CI* confidence interval; *HHF* hospitalization for heart failure, *MACE* major adverse cardiovascular events, *HR* hazard ratio, *MI* myocardial infarction, *NA* not applicable, *NR* not reported, *SCORED* Effect of Sotagliflozin on Cardiovascular and Renal Events in Patients with Type 2 Diabetes and Moderate Renal Impairment Who Are at Cardiovascular Risk, *SGLT2* sodium-glucose cotransporter 2

### Association of SGLT2i with safety outcomes among participants with diabetic kidney disease

**Fig. 2:**
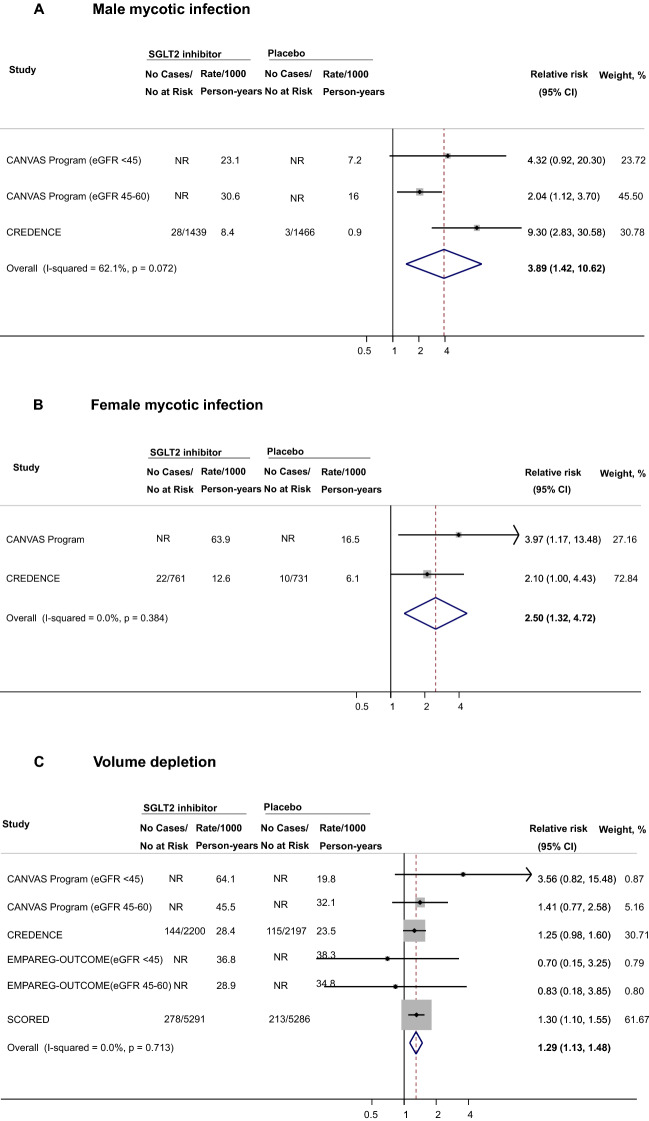

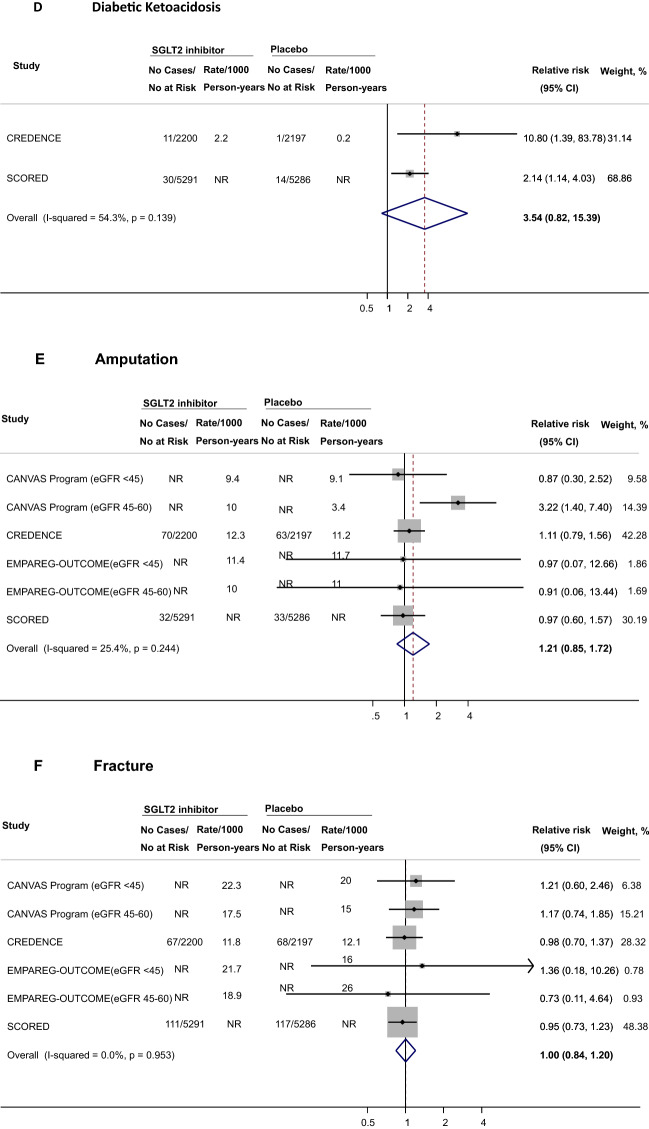

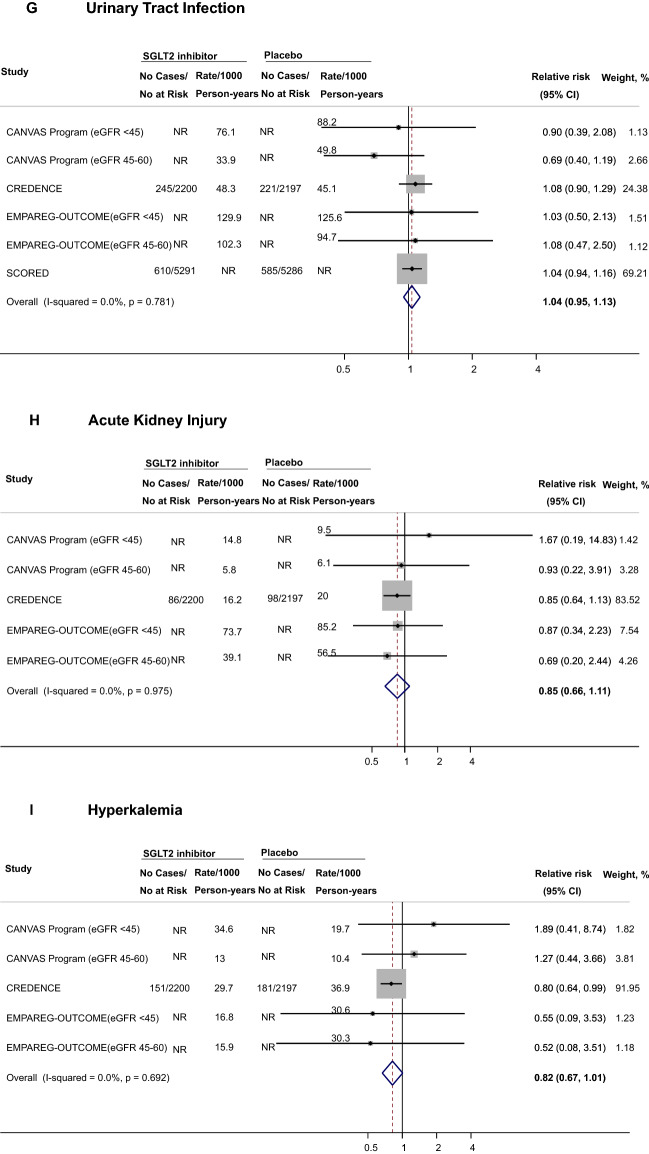
Effects of SGLT2 inhibitors on safety outcomes among individuals with diabetic kidney disease. CANVAS indicates Canagliflozin Cardiovascular Assessment Study, *CI* confidence interval, *CREDENCE* Canagliflozin and Renal Events in Diabetes with Established Nephropathy Clinical Evaluation, *eGFR* estimated glomerular filtration rate, *EMPA‐REG OUTCOME* Empagliflozin Cardiovascular Outcome Event Trial in Type 2 diabetes Mellitus Patients, *NR* not reported; *SCORED* Effect of Sotagliflozin on Cardiovascular and Renal Events in Patients with Type 2 Diabetes and Moderate Renal Impairment Who Are at Cardiovascular Risk; SGLT2, sodium-glucose cotransporter 2

A total of 4 trials reported data on safety outcomes among participants with DKD. SGLT2i were associated with higher risks of male mycotic infections (RR 3.89, 95% CI 1.42–10.62, n = 4,091 participants across 2 studies, Fig. 2A), female mycotic infections (RR 2.50, 95% CI 1.32–4.72, n = 2,100 participants across 2 studies, Fig. 2B), and volume depletion (RR 1.29, 95% CI 1.13–1.48, n = 18,832 participants across 4 studies, Fig. 2C). With respect to DKA, the effect estimate was increased but its CI was wide (RR 3.54, 95% CI 0.82–15.39, n = 14,974 participants across 2 studies, Fig. 2D). No association was observed for amputations (RR 1.21, 95% CI 0.85–1.72, n = 18,832 participants across 4 studies, Fig. 2E), bone fractures (RR 1.00, 95% CI 0.84–1.20, n = 18,832 participants across 4 studies, Fig. 2F), and UTI (RR 1.04, 95% CI 0.95–1.14, n = 18,832 participants across 4 studies, Fig. 2G). The RRs were 0.85 for AKI (95% CI 0.66–1.11, n = 8255 participants across 3 studies, Fig. 2H) and 0.82 for hyperkalemia (95% CI 0.67–1.01, n = 8255 participants across 3 studies, Fig. 2I). Overall, confidence intervals were wide for safety outcomes, limiting the precision of point estimates (Table 4).

**Table 4:** Effect of SGLT2 Inhibitors on safety events among patients with diabetic kidney disease

Outcome	No studies	No events	Sample size	RR (95% CI)	*I*^2^, %	*P*_Heterogeneity_	*P*_Egger test_
Male genital mycotic infections	2	98	4091	**3.89 (1.42–10.62)**	62.1	0.072	0.392
Female genital mycotic infections	2	53	2100	**2.50 (1.32–4.72)**	0.0	0.384	NA
Diabetic ketoacidosis	2	56	14,974	3.54 (0.82–15.39)	54.3	0.139	NA
Volume depletion	4	1016*	18,832	**1.29 (1.13–1.48)**	0.0	0.713	0.936
Amputations	4	248*	18,832	1.21 (0.85–1.72)	25.4	0.244	0.767
Bone fractures	4	475*	18,832	1.00 (0.84–1.20)	0.0	0.953	0.447
Urinary tract infections	4	1739*	18,832	1.04 (0.95–1.14)	0.0	0.781	0.339
Acute kidney injury	3	197*	8255	0.85 (0.66–1.11)	0.0	0.975	0.535
Hyperkalemia	3	359*	8255	0.82 (0.67–1.01)	0.0	0.692	0.601

Bold values indicate statistically significant estimates

*The number of events from the EMPA-REG OUTCOME trial were not reported and therefore not included in the table for the following outcomes: volume depletion, amputations, fractures, urinary tract infection, acute kidney injury, and hyperkalemia. CI indicates confidence interval, *EMPA‐REG OUTCOME* Empagliflozin Cardiovascular Outcome Event Trial in Type 2 diabetes Mellitus Patients; *I*^2^, I-squared, *RR* relative risk, *SGLT2* sodium-glucose cotransporter 2

## Discussion

We conducted a meta-analysis of several clinical outcomes of SGLT2i focused entirely on individuals with DKD. In a large sample of adults with T2D and DKD, we found that SGLT2i reduced the risks of MACE, kidney composite outcomes, HHF, cardiovascular death, nonfatal and fatal MI, nonfatal and fatal stroke, as well as all-cause mortality. Similar trends were observed when the sample was restricted to participants with reduced eGFR and those with moderate or severe albuminuria. The risks of mycotic infections, as well as volume depletion were greater among participants receiving SGLT2i compared to those on placebo.

Our findings add to previously published meta-analyses on SGLT2i by focusing exclusively on patients with DKD, and by including the most recent trials. Furthermore, our review is unique in its evaluation of clinical outcomes by eGFR and albuminuria levels and the assessment of the effects of SGLT2i on adverse safety events in participants with DKD. Additionally, we chose a random-effects meta-analysis approach as opposed to fixed-effects models used in prior meta-analyses [32, 33], due to the heterogeneity of patient populations included across the clinical trials of SGLT2i.

Our study confirms that the beneficial effects of SGLT2i on the three-point composite of MACE (nonfatal MI, nonfatal stroke, and cardiovascular death) extends to people with DKD. These findings corroborate results from a prior meta-analysis of four SGLT2i trials which found that SGLT2i reduced the MACE composite irrespective of baseline atherosclerotic CVD, HF, or kidney function status [33]. Likewise, a recent meta-analysis of five trials observed a beneficial effect of SGLT2i against MACE in patients with T2D [34]. In our meta-analysis, the protective association of SGLT2i with MACE persisted in subgroup analyses by baseline eGFR; a similar trend was noted among participants with severely increased albuminuria. Among participants with moderately increased albuminuria, the effect of SGLT2i on MACE remained protective although with less precise estimates, likely owing to the small number of studies in this subgroup analysis.

In our meta-analysis, SGLT2i provided the highest risk reduction for kidney composite outcomes and HHF compared to placebo. We found a 34% reduction in the risk of kidney composite outcomes. This finding was consistent across kidney function levels and in patients with moderate or severe albuminuria. A possible mechanism for this association relates to the role of SGLT2i in reducing the risk of sustained eGFR decline or ESKD [35]. Our finding is consistent with current guidelines recommending the use of SGLT2i among patients with T2D at moderate kidney failure risk [35].

Our observation of a beneficial effect of SGLT2i on HHF in patients with DKD is consistent with prior published meta-analyses of individuals with T2D [33, 34]. The benefits of SGLT2i on HHF risk applied widely across the drug class and were consistent across various eGFR and albuminuria levels. These findings support current guidelines recommending the use of SGLT2i in patients with or at high risk of HF, independent of glycemic considerations. HF occurs at higher rates among patients with CKD [36] and diabetes [37]. Albuminuria and reduced eGFR compound the risks of HHF and death [36]. The prevention of HHF may reduce the risk of ESKD and death in patients with CKD [38].

We found a protective effect of SGLT2i on the risk of stroke in patients with DKD. This finding confirms that from two recent meta-analyses [32, 33], although the two prior studies included only four studies in their stroke meta-analysis and implemented fixed-effect meta-analysis models, an analytical approach that assumes the presence of one true effect size underlying all the studies in the meta-analysis. We chose to conduct random-effects meta-analyses as this approach is more conservative and allows for between-study heterogeneity [27]. A recent meta-analysis of the effect of SGLT2i on the risk of stroke in patients with DKD observed a null association using the random-effects models, likely due to low statistical power [15]. The exact mechanisms for the beneficial effect of SGLT2i on stroke risk observed in our study is unclear, but may be mediated by prevention of atrial fibrillation (AF) and atrial flutter (AFL) [39]. Indeed, dapagliflozin was found to lower the risks of first AF/AFL as well as total AF/AFL events in a large sample of adults with T2D [40]. Two recent meta-analyses also found that SGLT2i were associated with a lower risk of AF [15, 41]. SGLT2i promote natriuresis, diuresis and thus may reduce the atrial diameter. Additionally, SGLT2i reduce cardiac remodeling, left ventricular mass, blood pressure, body weight, inflammation, oxidative stress and the sympathetic drive, all of which may promote AF/AFL, and thus increase the risk of stroke [42, 43].

With respect to safety outcomes, our review identified a knowledge gap as most trials were not powered to assess adverse effects of SGLT2i in the subgroup of patients with DKD, neither did they report safety information in this population. Additionally, data from the safety outcomes in this review were derived from a total of 2 to 4 studies. Consistent with prior reports, we found that the most commonly reported side effects of SGLT2i also extend to patients with DKD [6, 7, 11]. Indeed, in our meta-analysis, SGLT2i were associated with higher risks of genital mycotic infections, as well as volume depletion. Participants on SGLT2i were also at higher risk of DKA, although estimates were imprecise, likely owing to insufficient statistical power. It is unclear if participants who developed DKA in this review were on insulin. Although SGLT2i was linked to a higher risk of volume depletion, SGLT2i did not result in a higher risk of AKI in this meta-analysis. In fact, the risk of AKI appeared lower among those on SGLT2i. Prior evidence from observational studies has suggested that SGLT2i may be linked to a decreased risk of AKI [44–47]. While the exact mechanisms underpinning this association are unclear, possible hypotheses include the attenuation of ischemic-reperfusion injury to the kidney [48], as well as a reduction in tubular injury [49].

The public health and research implications of our findings are manifold. Our findings confirm the relevance of SGLT2i in the therapeutic scheme of patients with DKD. As SGLT2i prescription rates for patients with DKD are increasing [12], our findings highlight the importance of risk mitigation strategies in this population. These strategies include the proper hygiene of genitalia to prevent fungal infections, the proactive dose reduction of diuretics in euvolemic patients to prevent volume depletion, patient education on early recognition of DKA and implementation of STOP DKA protocol (stop SGLT2i, test for ketones, maintain intake of fluid and carbohydrates, and use maintenance and supplemental insulin) as well as foot examination to reduce the risk of amputations [35]. Further research is needed to determine the effect of SGLT2i among patients with advanced DKD. As most clinical trials focus on efficacy outcomes and are typically not powered to assess rare adverse safety effects of medications [50], additional studies using real-world data are needed to better evaluate the safety profile of SGLT2i in patients with DKD.

The limitations of this study should be acknowledged. First, most of the trials included in this meta-analysis excluded participants with eGFR less than 30 mL/min/1.73 m^2^; therefore, our findings may not be generalizable to individuals with advanced DKD. Second, the included SGLT2i trials did not always report the effect estimates for all the relevant outcomes specifically among participants with DKD (for example, the trials did not always report on individual kidney outcomes such as ESKD that would have been defined more consistently across trials); and we did not have access to individual participant data that could have been used to calculate the effect estimates for the unreported outcomes or perform meta-regression analyses. Likewise, the individual trials did not report data specifically in the subset of participants with albuminuria and eGFR ≥ 60 mL/min/1.73 m^2^; thus, we could not evaluate the effects of SGLT2i on outcomes in this subgroup. Third, the limited number of available studies limited our ability to perform meaningful subgroup analyses such as analyses by age, gender, or race/ethnicity, for example. Fourth, we defined DKD as the presence of CKD in individuals with T2D and the individual studies did not exclude causes of CKD other than T2D among the participants. Finally, the definition of the kidney composite outcomes was not consistent across the trials. These limitations notwithstanding, our review has several strengths. First, this is a comprehensive review of all information available to date on the efficacy and safety outcomes of SGLT2i in patients with DKD. Second, we used random-effects meta-analysis models, a more conservative approach which makes less assumptions about the effect of SGLT2i across included trials. Third, only placebo-controlled RCTs with low risk of bias were included. Fourth, we appraised the quality of studies using a standard quality assessment tool. Finally, our review is quantitative and highlights the magnitude of the associations between SGLT2i and various clinical outcomes.

## Conclusions

In conclusion, our findings confirm the beneficial effects of the SGLT2i class on cardiovascular and kidney outcomes, as well as mortality in patients with T2D and DKD. Our data support the current recommendations to prioritize the prescriptions of SGLT2i in patients with DKD, independent of glycemic control [35]. Further research is warranted to explore the effects of SGLT2i in patients with advanced DKD and to better characterize the safety profile of SGLT2i in patients with DKD.

## Supplementary Information

**Additional file 1**: **Fig S1.** Selection of studies for inclusion in the meta-analysis. **Fig S2.** Effects of SGLT2 Inhibitors on fatal and nonfatal stroke among individuals with Diabetic Kidney disease. **Table S1.** Definition of kidney composite outcomes across included trials. **Table S2.** Characteristics of Clinical Trials Included in the Meta-analysis. **Table S3.** Risk of Bias Assessment.

## Data Availability

The data that support the findings of this study are publicly available and included in this article and in its additional file.

## Abbreviations

AF: Atrial fibrillation
AFL: Atrial flutter
AKI: Acute kidney injury
CANVAS: Canagliflozin Cardiovascular Assessment Study
CI: Confidence interval
CKD: Chronic kidney disease
CREDENCE: Canagliflozin and Renal Events in Diabetes with Established Nephropathy Clinical Evaluation
CVD: Cardiovascular disease
DAPA-CKD: Dapagliflozin and Prevention of Adverse Outcomes in Chronic Kidney Disease
DECLARE‐TIMI 58: Dapagliflozin Effect on Cardiovascular Events–Thrombolysis in Myocardial Infarction 58
DKA: Diabetic ketoacidosis
DKD: Diabetic kidney disease
eGFR: Estimated glomerular filtration rate
EMPA‐REG OUTCOME: Empagliflozin Cardiovascular Outcome Event Trial in Type 2 diabetes Mellitus Patients
ESKD: End-stage kidney disease
HHF: Hospitalizations for heart failure
HR: Hazard ratio
MACE: Major adverse cardiovascular event
MI: Myocardial infarction
PRISMA: Preferred Reporting Items for Systematic Reviews and Meta-Analyses
RCT: Randomized controlled trial
RR: Relative risk
SCORED: Effect of Sotagliflozin on Cardiovascular and Renal Events in Patients with Type 2 Diabetes and Moderate Renal Impairment Who Are at Cardiovascular Risk
SGLT2i: Sodium-glucose cotransporter 2 inhibitor
SOLOIST-WHF: Effect of Sotagliflozin on Cardiovascular Events in Patients with Type 2 Diabetes Post Worsening Heart Failure
T2D: Type 2 diabetes
UACR: Urine albumin-to-creatinine ratio
UTI: Urinary tract infection
VERTIS CV: Evaluation of Ertugliflozin Efficacy and Safety Cardiovascular Outcomes Trial
