# Supplementary material for: Association of SGLT2 inhibitors with cardiovascular, kidney, and safety outcomes among patients with diabetic kidney disease: a meta-analysis
Source: Cardiovasc Diabetol. 2022 Mar 23;21:47. doi: 10.1186/s12933-022-01476-x (PMC9491404; doi:10.1186/s12933-022-01476-x)
Supplement: Supplementary file 1 — Additional file 1: Fig S1. Selection of studies for inclusion in the meta-analysis. Fig S2. Effects of SGLT2 Inhibitors on fatal and nonfatal stroke among individuals with Diabetic Kidney disease. Table S1. Definition of kidney composite outcomes across included trials. Table S2. Characteristics of Clinical Trials Included in the Meta-analysis. Table S3. Risk of Bias Assessment. [file 12933_2022_1476_MOESM1_ESM.docx]

**Association of SGLT2 Inhibitors with Cardiovascular, Kidney, and Safety Outcomes among Patients with Diabetic Kidney Disease: A Meta-analysis**

**Running title:** SGLT2 Inhibitors in Diabetic Kidney Disease

**Authors:** Arnaud D. Kaze,^1^ Min Zhuo,^2,3,4,5^ Seoyoung C. Kim,^2,3^ Elisabetta Patorno,^2,3^ Julie M. Paik,^2,3,4,6^

**Affiliations:**

^1^ Department of Medicine, LifePoint Health, Danville, VA

^2^ Division of Pharmacoepidemiology and Pharmacoeconomics, Department of Medicine, Brigham and Women’s Hospital, Boston, MA

^3^ Harvard Medical School, Boston, MA

^4^ Division of Renal (Kidney) Medicine, Department of Medicine, Brigham and Women’s Hospital, Boston, MA

^5^ Division of Nephrology, Department of Medicine, Beth Israel Deaconess Medical Center, Boston, MA

^6^ New England Geriatric Research Education and Clinical Center, VA Boston Healthcare System, Boston, MA

**Correspondence to:**

Julie Paik, MD, ScD, MPH

Brigham and Women’s Hospital

Division of Pharmacoepidemiology and Pharmacoeconomics

1620 Tremont Street, Suite 3030, Boston, MA, 02120

[jmpaik@bwh.harvard.edu](mailto:jmpaik@bwh.harvard.edu)

Phone: (617) 525-3423

**Table S1. Definition of kidney composite outcomes across included trials**

| CANVAS Program | composites of end-stage kidney disease, renal death, and either 40% decrease in eGFR or doubling of serum creatinine. |
| --- | --- |
| CREDENCE | composite of end-stage kidney disease (dialysis for at least 30 days, kidney transplantation, or eGFR <15 ml/min/1.73 m^2^ sustained for at least 30 days), doubling of serum creatinine, or death from renal cause. |
| DECLARE-TIMI 58 | composites of 40% decrease in eGFR, end-stage kidney disease, renal death. |
| DAPA-CKD | composites of 50% decrease in eGFR, end-stage kidney disease, death from renal or cardiovascular causes. |
| SCORED | composites of 50% decrease in eGFR, end-stage kidney disease (long-term dialysis, kidney transplantation or sustained eGFR <15mL/min/1.73m^2^ for ≥ 30 days). |

**Table S2. Characteristics of Clinical Trials Included in the Meta-analysis**

| **Study** | **SGLT2 inhibitor** | **No. with DKD** | **Age, y** | **Women,**  **%** | **White, %** | **Follow-up, y** | **CKD**  **Definition^*^** | **eGFR^*^**  **Range** | **HbA_1C_,**  **%** | **Diabetes duration, y** | **CVD,**  **%** | **HF,**  **%** |
| --- | --- | --- | --- | --- | --- | --- | --- | --- | --- | --- | --- | --- |
| EMPA-REG OUTCOME^6,18^ | Empagliflozin | 2250 | 66.0 (8.5) | 29.7 | 72.3 | 3.1 | eGFR<60 and/or UACR ≥ 300 | 30-59 | 8.1  (0.9) | 68.9% >10 y | 100 | 14.1 |
| CANVAS  Program^11,15,19,20^ | Canagliflozin | 2039 | 67.2 (7.6) | 41.8 | 82.1 | 2.4 | eGFR < 60 | 30-59 | 8.2  (1.0) | 16.0 (8.3) | 79.7 | 17.9 |
| DECLARE-  TIMI 58^7^ | Dapagliflozin | 1265 | 63.9 (6.8) | 37.4 | 79.6 | 4.2 | eGFR < 60 | NR | 8.3  (1.2) | 11.8 (7.8) | 40.6 | 10.0 |
| CREDENCE^8,21,22^ | Canagliflozin | 4401 | 63.0 (9.2) | 33.9 | 66.6 | 2.6 | eGFR 30-89 and UACR ≥ 300 | 30-89 | 8.3  (1.3) | 15.8 (8.6) | 50.4 | 14.8 |
| DAPA-CKD^9,23^ | Dapagliflozin | 2906 | 61.8 (12.1) | 33.1 | 53.2 | 2.4 | eGFR 25-75 and UACR ≥ 200 | 25-75 | NR | NR | 37.4 | 10.9 |
| VERTIS CV^10,24^ | Ertugliflozin | 1807 | 64.4 (8.1) | 30.0 | 87.8 | 3.0 | eGFR < 60 | 30-59 | 8.2  (1.0) | 13.0 (8.3) | 100 | 23.7 |
| SCORED^16^ | Sotagliflozin | 10584 | 69  (63-74) | 44.9 | 82.7 | 1.3 | eGFR < 60 | 25-59 | 8.3  (7.6-9.4) | NR | 48.6 | 31.0 |
| SOLOIST-  WHF^30^ | Sotagliflozin | 854 | 69  (63-76) | 33.3 | 93.2 | 0.8 | eGFR < 60 | 30-59 | 7.1  (6.4-8.3) | NR | NR | 100 |

CANVAS indicates Canagliflozin Cardiovascular Assessment Study; CKD, chronic kidney disease; CREDENCE, Canagliflozin and Renal Events in Diabetes with Established Nephropathy Clinical Evaluation; CVD, cardiovascular disease; DAPA-CKD, Dapagliflozin and Prevention of Adverse Outcomes in Chronic Kidney Disease; DECLARE‐TIMI 58, Dapagliflozin Effect on Cardiovascular Events–Thrombolysis in Myocardial Infarction 58; DKD, diabetic kidney disease; eGFR, estimated glomerular filtration rate; EMPA‐REG OUTCOME, Empagliflozin Cardiovascular Outcome Event Trial in Type 2 diabetes Mellitus Patients; HbA_1C_, hemoglobin A_1C_; HF, heart failure; NR, not reported; SCORED, Effect of Sotagliflozin on Cardiovascular and Renal Events in Patients with Type 2 Diabetes and Moderate Renal Impairment Who Are at Cardiovascular Risk; SGLT2, sodium-glucose cotransporter 2; SOLOIST-WHF, Effect of Sotagliflozin on Cardiovascular Events in Patients with Type 2 Diabetes Post Worsening Heart Failure; VERTIS CV, Evaluation of Ertugliflozin Efficacy and Safety Cardiovascular Outcomes Trial; UACR, urine albumin-creatinine ratio; y, years.

^*^ eGFR is reported in mL/min/1.73m^2^; UACR is reported in mg/g.

**Table S3. Risk of Bias Assessment**

|  | **Random sequence generation** | **Allocation**  **sequence**  **concealment** | **Blinding of participants**  **and personnel** | **Blinding of**  **outcome**  **assessment** | **Incomplete**  **outcome data**  **addressed** | **Selective**  **reporting** |
| --- | --- | --- | --- | --- | --- | --- |
| EMPA-REG OUTCOME | Low | Low | Low | Low | Low | Low |
| CANVAS  Program | Low | Low | Low | Low | Low | Low |
| DECLARE-  TIMI 58 | Low | Low | Low | Low | Low | Low |
| CREDENCE | Low | Low | Low | Low | Low | Low |
| DAPA-CKD | Low | Low | Low | Low | Low | Low |
| VERTIS CV | Low | Low | Low | Low | Low | Low |
| SCORED | Low | Low | Low | Low | Low | Low |
| SOLOIST-  WHF | Low | Low | Low | Low | Low | Low |

176 records identified through database searches

176 records screened after duplicates removed

153 records excluded

23 Full-text articles assessed for eligibility

15 full-text articles excluded

no data on outcomes of interest (n=8)

not RCT (n=3)

no data on patients with DKD (n=4)

Trials included (n = 8)

Included

Eligibility

Identification

Screening

**Figure S1. Selection of studies for inclusion in the meta-analysis.**

DKD indicates diabetic kidney disease; RCT, randomized controlled trial.


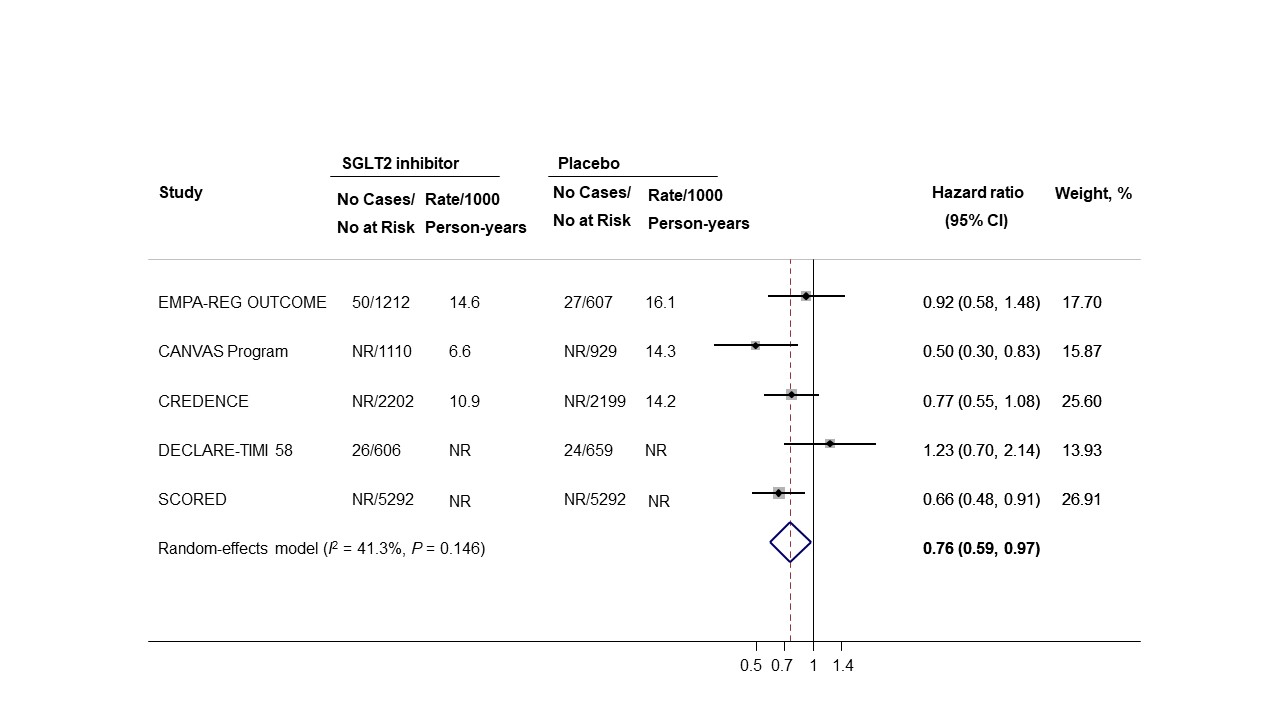


**Figure S2. Effects of SGLT2 Inhibitors on fatal and nonfatal stroke among individuals with Diabetic Kidney disease.**

CANVAS indicates Canagliflozin Cardiovascular Assessment Study; CI, confidence interval; CREDENCE, Canagliflozin and Renal Events in Diabetes with Established Nephropathy Clinical Evaluation; DECLARE‐TIMI 58, Dapagliflozin Effect on Cardiovascular Events–Thrombolysis in Myocardial Infarction 58; EMPA‐REG OUTCOME, Empagliflozin Cardiovascular Outcome Event Trial in Type 2 diabetes Mellitus Patients; *I*^2^, I-squared; NR, not reported; SCORED, Effect of Sotagliflozin on Cardiovascular and Renal Events in Patients with Type 2 Diabetes and Moderate Renal Impairment Who Are at Cardiovascular Risk; SGLT2, sodium-glucose cotransporter 2.
